# The Role of Pharmacogenetics in the Effectiveness of Rheumatoid Arthritis Treatment with Leflunomide

**DOI:** 10.3390/genes17050573

**Published:** 2026-05-18

**Authors:** Paulina Plewa, Anna Jędrasiak, Oliwia Jerzyńska, Aleksandra Dach, Maria Domańska, Andrzej Pawlik

**Affiliations:** Department of Physiology, Pomeranian Medical University, 70-111 Szczecin, Poland; paulina.plewa@op.pl (P.P.); annajedrasiak99@gmail.com (A.J.); oliwia.w.jerzynska@gmail.com (O.J.); dach.aleksandra@icloud.com (A.D.); maria.domanska@onet.eu (M.D.)

**Keywords:** rheumatoid arthritis, leflunomide, teriflunomide

## Abstract

This article discusses rheumatoid arthritis (RA) as a chronic, systemic autoimmune disease leading to progressive joint damage and multi-organ complications. The complex pathogenesis of the disease is presented, involving the interaction of environmental, genetic, and immunological factors, including the role of autoantibodies and proinflammatory cytokines. Particular attention is paid to leflunomide, a disease-modifying antirheumatic drug (DMARD), which primarily works by inhibiting the DHODH enzyme, leading to reduced T and B cell proliferation. The additional anti-inflammatory properties of the drug’s active metabolite, teriflunomide, and its impact on signaling pathways related to the immune response are also discussed. This article examines the variability in patient responses to leflunomide treatment in terms of both efficacy and toxicity, with particular emphasis on the potential role of pharmacogenetic factors. It was pointed out that polymorphisms in genes related to drug metabolism, transport, and mechanism of action may influence the pharmacokinetics and safety of the therapy. It was also emphasized that the available data are primarily derived from observational studies and small cohorts, and the results are often inconsistent. Although some genetic variants and plasma teriflunomide concentrations show potential as predictors of treatment response, the current level of evidence does not support the routine use of pharmacogenetic testing in clinical practice. The article emphasizes that the pharmacogenetics of leflunomide represents a promising, yet still exploratory, avenue of research in the context of personalized RA therapy. It emphasizes the need for larger, well-designed clinical trials and the development of standardized guidelines, which would be necessary before the potential implementation of such strategies in routine clinical practice.

## 1. Introduction

Rheumatoid arthritis (RA) is a chronic, systemic autoimmune disease that results in inflammatory infiltration of the synovial membrane, leading to progressive joint degradation and multisystem complications. It affects 0.5–2.0% of the global population and it constitutes a significant public health problem [1,2,3,4].

Although the exact causes of the disease remain unknown, RA has been associated with environmental factors and genetic predisposition. Human leukocyte antigens (HLAs) play a key role in this regard. A higher risk of the disease has been observed with specific alleles of the HLA-DRB1 gene (especially *04 and *10) [1,5].

It is believed that the pathogenesis of RA is based on an immunological cascade occurring within the synovial membrane and joint fluid [1]. Environmental factors are thought to promote protein citrullination [6], which is then targeted by autoantibodies (anti-citrullinated protein antibodies) [2]. The resulting immune complexes induce activation of synovial macrophages, which release pro-inflammatory cytokines (TNF-α, IL-1, IL-6), leading to progressive osteoclastogenesis and, consequently, bone erosion [1,2].

Genetic factors also influence how patients with RA respond to medications. The aim of this article was to review the genetic factors that influence the efficacy of leflunomide treatment in patients with RA. The literature search was conducted in two databases: PubMed and Scopus. Publications published between 1998 and 2026 were included. Combinations of keywords such as “leflunomide,” “polymorphism,” “rheumatoid arthritis,” “therapy,” “dihydroorotate dehydrogenase” and “treatment” were used to identify relevant publications. The most important publications were selected based on their relevance to the current state of knowledge, methodological quality, and direct relevance to the pharmacogenetics of leflunomide and the treatment of RA.

The primary goal of treating RA is to control inflammation, i.e., to achieve disease remission and thereby improve long-term quality of life. Therapeutic strategies include non-pharmacological care, pharmacological care, and, if pharmacological treatment fails, surgical intervention [7]. The most important pharmacological approach in RA is the use of disease-modifying anti-rheumatic drugs (DMARDs). Their introduction immediately after diagnosis is recommended [8]. The table below shows the classification of DMARDs (Table 1).

Conventional synthetic DMARDs (csDMARDs) are recommended as first-line therapies. Targeted synthetic DMARDs (tsDMARDs) and biologic DMARDs (bDMARDs) are used as second-line therapies in cases of failure or inadequate response to csDMARDs.

The most common csDMARDs include methotrexate, sulfasalazine, hydroxychloroquine, and leflunomide [9]. This group of drugs has different mechanisms of action. In this article, we focus on the pharmacogenetic effectiveness of leflunomide.

Leflunomide is a csDMARD primarily used in the treatment of RA and is also indicated in psoriatic arthritis, where it delays joint damage caused by inflammation. However, it does not significantly improve skin symptoms in psoriatic arthritis [1,10,11]. It exhibits immunomodulatory effects by reducing the number of activated T lymphocytes [12]. It is an isoxazole derivative and is metabolised primarily in the liver and intestines [13].

Leflunomide therapy typically involves a 100-mg loading dose for the first 3 days. This dose carries a higher risk of adverse events and is therefore recommended only for a selected group of patients. Subsequently, maintenance doses of 10 mg or 20 mg are administered. The maintenance dose should be individually adjusted depending on the patient’s tolerance [11]. It has been reported that 20–40% of patients discontinue leflunomide therapy because of drug toxicity [14]. Abdominal pain, diarrhoea, nausea, vomiting, rash, pruritus, and alopecia are among the symptoms leading to discontinuation. However, one of the most serious adverse effects is hepatotoxicity, including significant elevation of liver parameters [11,14,15,16]. The occurrence of adverse events is influenced by high interindividual variability in teriflunomide plasma concentrations; therefore, drug levels should be monitored during therapy [11]. This variability, and thus the clinical response, may be determined by several factors. The stage of RA is an important consideration when selecting leflunomide therapy. There are also reports of the influence of enterohepatic circulation and significant genetic factors affecting enzymatic expression and, consequently, teriflunomide metabolism [14,16,17,18]. Such high interindividual variability in leflunomide treatment suggests a strong need for personalised therapy.

Various tools can be used to optimise therapy with a given pharmaceutical. One such tool is the application of pharmacogenetic knowledge. Pharmacogenetics examines the relationship between genes and patient response to treatment. Applying this knowledge in practice allows for the selection of appropriate therapy, increasing the likelihood of achieving the desired therapeutic effect. It also reduces the risk of severe adverse effects by identifying patients at higher risk of such reactions [19]. This approach supports decisions regarding individualised therapy, improving not only treatment effectiveness but also, above all, patient safety.

The aim of this article is to present current knowledge on the pharmacogenetics of leflunomide in the treatment of RA, with particular emphasis on the influence of genetic factors on the efficacy and safety of therapy. This includes assessing the potential of identifying genetic polymorphisms to individualise treatment, optimise dosing, and reduce the risk of adverse events, as well as demonstrating the potential for implementing a personalised approach in clinical practice.

## 2. Leflunomide—Mechanism of Action and Pharmacokinetics

### 2.1. Biochemical Mechanism of Action

The primary mechanism of action of leflunomide is the inhibition of the mitochondrial enzyme dihydroorotate dehydrogenase (DHODH). This enzyme catalyses the oxidation of dihydroorotate to orotate, representing a crucial step in the de novo pyrimidine synthesis pathway. Inhibition of this enzyme results in reduced synthesis of uridine monophosphate. Consequently, this leads to cell cycle arrest in the G1 phase via a mechanism dependent on p53 protein activity. Activated T and B lymphocytes are particularly sensitive to this effect because they have an increased requirement for pyrimidines [20,21,22].

### 2.2. Role of the Active Metabolite A77 1726

A77 1726 (teriflunomide) is the active metabolite of leflunomide responsible for its pharmacological effects. The primary mechanism of action, observed at relatively lower concentrations of the metabolite, is inhibition of DHODH. At higher concentrations, it also affects tyrosine kinase-dependent mechanisms, inhibiting early activation of T and B cells [20,23].

Studies investigating the effects of A77 1726 in RA have identified additional mechanisms of action. The effect of A77 1726 on the activation of MAPK kinases in synovial fibroblasts stimulated with IL-1β has been analysed. Effective inhibition of JNK1/2 activation and partial inhibition of ERK1/2 and p38 activation were observed. As a result, a significant reduction in the secretion of certain matrix metalloproteinases produced by IL-1β-stimulated fibroblasts was noted [24].

**Figure 1:**
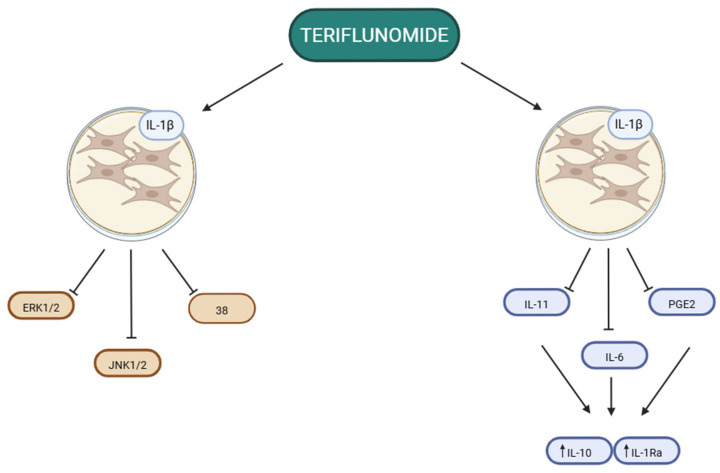
The effect of teriflunomide on the signaling pathways and secretory profile of synovial fibroblasts in RA. Studies conducted on IL-1β-stimulated fibroblast cultures showed that teriflunomide inhibits JNK1/2 and partially ERK1/2 and p38. Furthermore, this metabolite modulates the inflammatory response by reducing the levels of proinflammatory cytokines (IL-11, IL-6, PGE2) and increasing the levels of anti-inflammatory factors (IL-10, IL-1Ra). Created in BioRender. Plewa, P. (2026) https://BioRender.com/j72i91x (accessed on 13 April 2026).

A77 1726 also modulates cytokine levels. In in vitro studies, at a drug concentration of 100 µM, a decrease in pro-inflammatory factors such as IL-11, IL-6, and prostaglandin E2 was observed, accompanied by an increase in the levels of IL-10 and the IL-1 receptor antagonist, both of which have anti-inflammatory effects. Part of this effect appears to be related to inhibition of p38 kinase activation, a member of the MAPK family, by A77 1726 (Figure 1) [25].

Under in vitro conditions, the JAK/STAT pathway is also modulated by the active metabolite A77 1726, leading to a reduction in TNF and IL-17 production through inhibition of STAT6 phosphorylation. This effect persists even after uridine administration, indicating that the mechanism of action is independent of DHODH inhibition [26].

Leflunomide also affects systemic parameters in patients with RA. Studies in animal models have demonstrated a potential mechanism by which leflunomide, or more precisely its active metabolite teriflunomide (A77 1726), may reduce the risk of cardiovascular events. Analysis of samples revealed modulation of gene expression related to lipid metabolism, accompanied by increased phosphorylation of AMPKα and acetyl-CoA carboxylase. As a result, reduced lipid accumulation in liver cells and improved endothelial function were observed. This may be due to activation of the AMPK pathway, leading to inhibition of lipid synthesis. Additionally, the drug’s general mechanism of action, involving DHODH inhibition, contributes to reducing inflammation and improving cellular function [27].

The broad spectrum of action of A77 1726 suggests that its concentration may be critical for treatment efficacy. Analysis of Disease Activity Score in 28 joints using the Erythrocyte Sedimentation Rate (DAS28-ESR) scores showed that patients with A77 1726 concentrations higher than 10 µg/mL were more likely to achieve low disease activity or remission. These findings suggest that A77 1726 concentration may serve as a predictor of response to leflunomide treatment [28].

The data presented above indicate the multifaceted action of A77 1726, encompassing both immunological and metabolic mechanisms, which translates into its efficacy in the treatment of RA.

### 2.3. Pharmacokinetics

Leflunomide is characterised by high bioavailability following oral administration and is almost entirely converted into its active form, A77 1726. Its metabolism occurs primarily in hepatic microsomes via cytochrome P450 enzymes (mainly CYP1A2 and CYP2C19). Studies indicate that polymorphisms in these enzymes may affect plasma concentrations of the metabolite [29]. A77 1726 is more than 99% bound to plasma proteins, and its free form, responsible for the clinical effect, accounts for less than 1%. There is a strong correlation between the total drug concentration and the free metabolite, which allows estimation of the biologically active fraction [30]. The active metabolite has a relatively long half-life of approximately 2 weeks, allowing it to persist in the body for many days. Pharmacokinetic studies have shown that the metabolite reaches a maximum plasma concentration of about 2 µg/mL. It is also characterised by high total drug exposure (area under the curve of approximately 470–480 µg·h/mL), and its concentration remains detectable for up to 624 h after administration [31].

The active metabolite A77 1726 is excreted both unchanged and as glucuronides—approximately 43% in urine and 48% in faeces. Because of enterohepatic circulation, administration of substances that interfere with this process may accelerate drug elimination [32].

Studies comparing mean A77 1726 concentrations with disease activity have shown that higher metabolite levels are associated with a more favourable clinical response in patients, suggesting the potential value of individualising the drug dosage [33].

### 2.4. Factors Influencing Plasma Metabolite Levels

Given the demonstrated correlation between A77 1726 concentrations and treatment efficacy, it is important to identify the factors influencing variability in its plasma levels. Previous studies have shown that sex, age, and body weight have no clinically significant effect on the pharmacokinetics of A77 1726 or on the need for dose adjustment [32,33].

Among comorbidities, particular attention is given to renal and hepatic dysfunction. Pharmacokinetic data from the FDA report on leflunomide (not directly A77 1726) indicate that renal impairment (creatinine clearance of <30 mL/min) does not cause clinically significant changes in the drug’s pharmacokinetics. Clinical studies evaluating A77 1726 concentrations have also shown that patients undergoing haemodialysis do not require dose adjustment [34,35]. Mild and moderate hepatic impairment do not warrant dose adjustment of leflunomide, whereas its use is contraindicated in severe hepatic impairment [34].

Pharmacokinetic models provide additional insight. A study using a semi-physiologically based pharmacokinetic model showed that the concentration of the active metabolite depends primarily on lean body mass and liver function. Although liver enzymes (CYP1A2 and CYP2C19) are involved in drug metabolism, in this model polymorphisms of these enzymes did not have a statistically significant effect on A77 1726 concentrations in the studied population. Previously published reports highlighting the importance of the free fraction in relation to clinical response were also noted [35].

## 3. Pharmacogenetics of Leflunomide

### 3.1. Genetic Polymorphisms Affecting Drug Metabolism

Leflunomide is a prodrug that undergoes biotransformation to its pharmacologically active metabolite, A77 1726, known as teriflunomide. Its activation is a multienzymatic process. Cytochrome P450 (CYP) enzymes catalyse the oxidative ring-opening of the isoxazole ring of leflunomide, leading to the formation of teriflunomide. CYP1A2 and CYP2C19 are primarily involved, whereas CYP3A4 and CYP2C9 play a supporting role. Polymorphisms in these cytochromes play an important role in leflunomide metabolism.

The following polymorphisms have been identified in the CYP2C19 gene: C-163A, C-729T, T-739G, and single-nucleotide polymorphisms such as the CYP2C19*2, *3, *4, and *17 alleles. Teriflunomide concentrations have been shown to differ among carriers of specific alleles. The CYP2C19*2 allele has been associated with higher teriflunomide levels and, consequently, a better response to treatment. However, no increase in adverse effects was observed [29]. On the other hand, another study identified a correlation between CYP2C19*2 carriers and a higher percentage of drug-related toxicity events. Moreover, CYP2C19 intermediate and poor metabolisers may not benefit from leflunomide therapy because they exhibit lower concentrations of its pharmacologically active metabolite [36,37].

The CYP1A2*1F allele is located in intron 1 of the CYP1A2 gene. It has been reported to play a significant role in enzymatic activity in smokers, whereas in non-smokers it does not appear to have the same impact [37]. This suggests a strong environmental influence on CYP1A2 function. CYP1A2*1F has also been identified as an allele associated with drug toxicity [38], particularly in carriers of the CYP1A2*1F C allele rather than the A allele [14].

Two main single-nucleotide polymorphisms of CYP2C9 have been described: CYP2C9*2 and CYP2C9*3. These alleles are associated with reduced enzyme activity and, consequently, impaired substrate metabolism. Although CYP2C9 is not a major contributor to leflunomide metabolism, a few case reports have described a potential association between the CYP2C9*3 allele and drug toxicity [39]. No functionally significant variants of the CYP3A4 gene have been identified.

### 3.2. ABC Transporters and Drug Distribution

Proteins from the ABC family are a group of membrane transporters involved in the transport of various molecules across cell membranes. One of these is ABCG2, which is predominantly expressed in the gastrointestinal tract and liver. ABCG2 is responsible for removing toxins and preventing their accumulation. It also transports many DMARDs, including leflunomide and its active metabolite [40]. Kim et al. [41] presented a study suggesting that the C allele of ABCG2 is associated with higher concentrations of teriflunomide. However, in a study conducted by Wiese et al. [37], the A allele of the ABCG2 gene was correlated with certain adverse effects. Furthermore, a mutation in the p53 gene may cause leflunomide resistance due to ABCG2 overexpression [42]. By contrast, Makarem et al. [43] found no significant association between ABCG2 polymorphisms and leflunomide efficacy. Certain single-nucleotide polymorphisms have also been detected in coding regions, but these do not appear to affect the function or expression of the transporters [44].

Another transporter involved in leflunomide distribution, although to a lesser extent, is ABCC2. Single-nucleotide polymorphisms in ABCC2 have been found to alter the pharmacokinetics of certain drugs. The c.1446C>G allele has been associated with reduced sensitivity to statins, likely due to accelerated excretion [45]. Furthermore, the 24C>T polymorphism has been linked to increased drug concentrations and, consequently, a higher risk of adverse effects [46].

However, no studies on leflunomide have been conducted to date. It may be assumed, however, that similar effects could occur.

Studies discussed above that examine the relationship between genetic polymorphisms and the efficacy of leflunomide have several serious limitations. Many are based on small sample sizes, limiting their statistical power. In addition, numerous analyses focus on single-gene polymorphisms and assess pharmacokinetics and pharmacodynamics separately. Furthermore, the metabolism of leflunomide is complex and involves both enzymatic and non-enzymatic pathways, which poses challenges in drawing definitive conclusions. Finally, in vitro studies may not accurately reflect the drug’s metabolism in vivo [36]. As a result, clinical applications of findings cited below are very limited and in need of further research.

### 3.3. Genetic Variants Related to Drug Targets

Teriflunomide inhibits DHODH, thereby suppressing pyrimidine synthesis and reducing lymphocyte proliferation. The human DHODH gene is highly conserved. A single-nucleotide polymorphism affecting the first exon of the coding region has been identified. A study conducted by Pawlik et al. [47] suggests that DHODH gene polymorphism may influence the efficacy of leflunomide treatment. Patients with the C allele showed a better response to the drug than carriers of the A allele. Despite the greater efficacy of leflunomide, carriers of the C allele may also be at risk of experiencing toxic effects of the drug. This observation does not appear to apply to patients with the A allele [40,48]. Moreover, response to leflunomide has been shown to correlate with haplotype 2 of the DHODH gene. This polymorphism is associated with increased teriflunomide concentrations and, consequently, higher drug efficacy [48].

**Figure 2:**
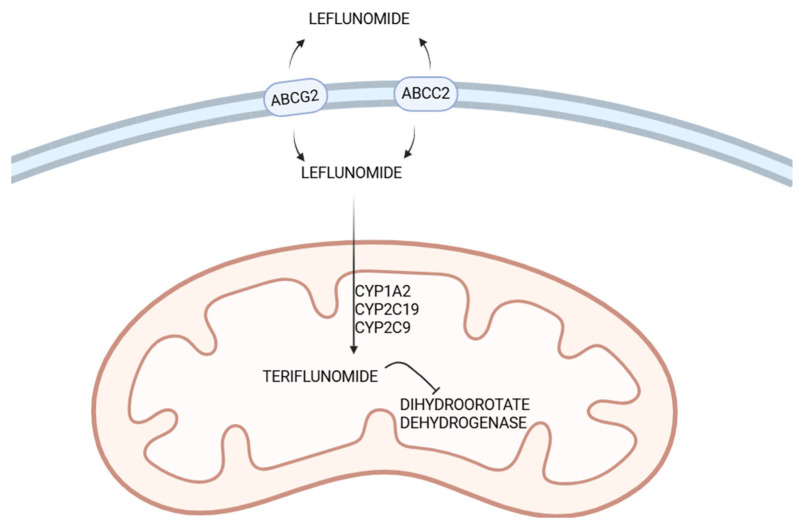
The role of ABC transporters in leflunomide pharmacokinetics and metabolism. ABCG2 and ABCC2 transporters are involved in the transport of leflunomide and its active metabolite. Teriflunomide is the active form of leflunomide and is responsible for its pharmacological action; at lower concentrations, its primary mechanism of action is inhibition of dihydroorotate dehydrogenase. Leflunomide metabolism is primarily mediated by CYP1A2 and CYP2C19, with CYP3A4 and CYP2C9 contributing to its metabolism. Their polymorphism may significantly impact the drug’s biotransformation. Created in BioRender. Dach, A. (2026) https://BioRender.com/dol3gzt (accessed on 13 April 2026).

Figure 2 provides a summary of leflunomide metabolism in cells, as described in Section 3.1, Section 3.2 and Section 3.3.

Because leflunomide affects lymphocyte proliferation and, consequently, the levels of cytokines they release, researchers have investigated whether polymorphisms in IL-1β, IL-6, and TNF-α influence treatment outcomes. These cytokines are responsible for modulating the pro-inflammatory response. TNF-α, IL-1, and IL-6 contribute to joint damage in patients with RA by promoting cartilage and matrix degradation. In addition, IL-6 induces B- and T-cell activation and the production of autoantibodies [49]. Pawlik et al. [50] observed no significant differences in leflunomide efficacy across different cytokine gene variants.

Table 2 summarises genetic polymorphisms and their role in leflunomide metabolism, as discussed above in Section 3.1, Section 3.2 and Section 3.3.

## 4. Clinical Evidence Linking Pharmacogenetic Variants with Treatment Outcomes

Clinical studies have indicated that variability in response to leflunomide in patients with RA is partially genetically determined. However, the available evidence remains heterogeneous and in some cases contradictory, which limits the ability to draw consistent conclusions. This variability involves genes related both to drug metabolism and to its mechanism of action [51]. Leflunomide is a prodrug whose active metabolite is A77 1726 (teriflunomide). The therapeutic effect is exerted by inhibiting dihydroorotate dehydrogenase (DHODH), a key enzyme in pyrimidine synthesis in lymphocytes [28]. Importantly, current evidence integrates both pharmacokinetic (drug exposure) and pharmacodynamic (drug target) variability, which should be considered separately when interpreting pharmacogenetic associations. One of the primary factors contributing to this variability is the presence of polymorphisms in drug-metabolising genes, particularly CYP2C19, which modulate exposure to the active metabolite [29]. Membrane transporters, such as ABCG2, also play a significant role by influencing the distribution and elimination of A77 1726, thereby contributing to interindividual differences in its concentration [41]. However, the strength and consistency of evidence for ABCG2 remain limited, and its direct impact on clinical outcomes has not been clearly established. In contrast, evidence for other transporters (e.g., ABCC2) is currently indirect and based on extrapolation from other drugs, and should therefore be interpreted cautiously.

The clinical response to leflunomide treatment is most commonly assessed using the DAS28 score and the ACR20/50/70 criteria [52,53]. Clinical studies have demonstrated that elevated concentrations of the active metabolite A77 1726 are associated with improved therapeutic responses and reduced disease activity, as measured by DAS28 (*p* < 0.001). This relationship reflects an exposure-response association rather than a direct genotype-response relationship. This highlights the pivotal role of drug exposure as a determinant of treatment efficacy [48,54,55]. Genetic factors influencing the pharmacokinetics of leflunomide also contribute to variability in treatment response. Polymorphisms in CYP2C19 have been associated with differences in the concentration and clearance of A77 1726, resulting in variability in systemic exposure among patients [29,37,38]. However, the direction and magnitude of this effect remain inconsistent across studies. Some reports indicate higher metabolite levels in poor metabolizers, whereas others show reduced exposure or no significant association. These discrepancies are likely due to differences in study design, population characteristics, and endpoint definitions rather than a single biological mechanism. However, pharmacogenetic data indicate that the effect of genotype on A77 1726 concentrations themselves remains limited and inconclusive. Moreover, genetic variability more often translates into differences in clinical response or toxicity than directly into pharmacokinetic changes [55,56]. For the DHODH gene, the molecular target of A77 1726, a significant association between specific haplotypes and disease activity has been demonstrated (β = 0.56, *p* = 0.01), highlighting the importance of pharmacodynamic mechanisms in modulating treatment response. This suggests that variability at the level of the drug target may be more relevant for clinical outcomes than variability in drug metabolism. At the same time, the same study confirmed an association between higher concentrations of A77 1726 and lower DAS28 scores (*p* < 0.001) [48]. The results of multigene analyses suggest an additional role for genes such as ABCG2, ESR1, ESR2, UMPS, and DPYD [10]. However, further research is required to determine their clinical significance. It is important to note that the majority of available studies are observational and involve relatively small patient groups, which limits the ability to definitively determine the utility of pharmacogenetic testing in routine clinical practice [51].

The impact of genetic polymorphisms on the risk of leflunomide-related adverse effects is less well understood than their role in treatment efficacy, and the available data remain limited. To date, no clear and reproducible associations have been demonstrated between specific genetic variants and the occurrence of adverse effects, such as hepatotoxicity or gastrointestinal disorders [39]. Some studies have indicated a potential correlation between variations in drug-metabolising genes, such as CYP2C19, and the safety profile of therapy. However, these findings have been inconsistent and have not been validated in independent cohorts [37]. Additionally, evidence suggests that elevated concentrations of A77 1726 may be associated with an increased risk of adverse effects, indicating that toxicity is more likely driven by systemic exposure than by genotype itself [12]. However, other studies have not confirmed a significant association between CYP2C19 polymorphisms and leflunomide toxicity, highlighting inconsistencies in the available data and the need for further research [37].

The prevalence of CYP2C19 polymorphisms varies significantly across populations. Although this provides a strong biological rationale for interethnic variability, direct clinical evidence linking these differences to treatment outcomes remains limited. Loss-of-function alleles are more common in Asian populations than in European populations, as widely documented in pharmacogenetic studies. These differences result in a higher prevalence of the poor metaboliser phenotype in Asian populations, which is relevant to the pharmacokinetics of drugs metabolised by CYP2C19 [57,58]. Research on leflunomide has shown that CYP2C19 variants influence the concentration and clearance of A77 1726. However, most analyses have been conducted in relatively homogeneous populations, limiting direct cross-ethnic comparisons [29]. Despite a clear biological rationale for ethnic differences, there is still a lack of conclusive clinical evidence directly linking these differences to the efficacy or safety of leflunomide treatment [43,51].

## 5. Pharmacogenetic Testing in Clinical Practice

The use of pharmacogenetics for leflunomide in clinical practice remains non-routine, and its application is currently limited to research settings because of the lack of conclusive clinical evidence. This is primarily due to inconsistent results, small study populations, and lack of standardized guidelines. Available pharmacogenetic evidence suggests that genetic variability influences treatment through multiple mechanisms, including pharmacokinetics (e.g., CYP2C19 and ABCG2), pharmacodynamic response (e.g., DHODH), and toxicity risk (e.g., CYP1A2 and CYP2C19), although the strength of evidence differs between these domains. [39,59,60]. The CYP1A2*1F polymorphism (CC genotype) has been significantly associated with an increased risk of leflunomide toxicity (odds ratio of approximately 9.7), indicating its potential role in identifying high-risk patients [38,61]. Additionally, the rs762551 variant in the CYP1A2 gene has been linked to an increased risk of treatment discontinuation due to adverse effects (hazard ratio of approximately 2.29), highlighting the importance of metabolic variability in treatment tolerance [14,62]. Moreover, CYP2C19 polymorphisms have been shown to influence the course of therapy, with patients exhibiting the poor metaboliser phenotype being more likely to discontinue treatment. This suggests the potential utility of these polymorphisms as markers of treatment safety. Genetic variation in the DHODH target gene has been associated with both treatment efficacy and the achievement of disease control, indicating its possible role as a biomarker of therapeutic response. Furthermore, both DHODH haplotypes and the concentration of the active metabolite (teriflunomide) have been shown to correlate with disease activity as measured by the DAS28 index, suggesting the potential to predict clinical response [43,54]. The relationship between teriflunomide concentration and clinical outcomes further supports the concept of individualised leflunomide dosing based on the patient’s pharmacogenetic and pharmacokinetic profile [48]. However, despite these associations, no single pharmacogenetic marker currently demonstrates sufficient predictive value for routine clinical decision-making, highlighting the need for integrative approaches combining genetic, pharmacokinetic, and clinical data. Before initiating clinical treatment with leflunomide in patients with RA, it is very important to assess the patients’ clinical parameters and disease activity indices. The patients’ response to prior treatment with medications such as methotrexate or sulfasalazine should also be taken into account. It is also important to evaluate which adverse effects occurred following the use of these medications. Only a comprehensive assessment of the patient’s clinical parameters, response to previously used medications, and any pharmacogenetic testing can help in selecting the appropriate medication for a patient with RA.

## 6. Conclusions

A growing body of evidence indicates that the efficacy and safety of leflunomide treatment in RA not only depend on its mechanism of action but are also significantly influenced by individual variability, including genetic and pharmacokinetic factors. The active metabolite teriflunomide (A77 1726) plays a particularly important role because its plasma concentration correlates with both therapeutic response and the risk of adverse events. Studies suggest that polymorphisms in genes related to drug metabolism (e.g., CYP2C19, CYP1A2), transport (ABCG2), and the molecular target (DHODH) may influence the course of therapy; however, the findings are often inconsistent and dependent on the studied population.

Clinical and experimental data highlight the potential of pharmacogenetics as a tool to support the personalisation of RA treatment, but most available studies are observational and involve relatively small patient groups. Consequently, their translation into routine clinical practice remains limited. An additional challenge is the lack of standardised methods for assessing treatment response, clearly defined biomarkers, and consistent guidelines for interpreting genetic test results. The complexity of leflunomide metabolism and the coexistence of multiple factors influencing therapeutic response—such as the patient’s clinical status, comorbidities, and drug interactions—also remain significant issues.

Therefore, future research should focus on three key areas:

Mechanistic studies—precisely defining the relationship between genetic variants and the pharmacokinetics and pharmacodynamics of leflunomide, including their impact on teriflunomide concentrations and immune response.

Clinical trial design—conducting large, prospective studies involving diverse patient populations, with long-term follow-up and standardisation of endpoints and response biomarkers.

Personalised treatment strategies—identifying predictors of response and toxicity to enable individualised dosing and therapy selection based on the patient’s genetic profile and pharmacokinetic parameters.

This approach may contribute to increased therapeutic efficacy, reduced adverse effects, and the implementation of personalised medicine in the treatment of RA. At the same time, it underscores the importance of further developing our understanding of the interactions between patient genotype, drug metabolism, and immune response, which collectively determine treatment outcomes.

## Figures and Tables

**Table 1:** Classification of DMARDs.

Category	Subcategory	Abbreviation	Examples	References
Synthetic DMARDs (sDMARDs)	Conventional	csDMARDs	leflunomide, methotrexate, sulfasalazine, hydroxychloroquine	[1,8]
	Targeted	tsDMARDs	JAK inhibitors (tofacitinib, baricitinib)	[1,8]
Biological DMARDs (bDMARDs)	Originator	boDMARDs	adalimumab, etanercept, rituximab	[1,8]
	Biosimilar	bsDMARDs	biosimilar versions of originator biologics	[1,8]

**Table 2:** Summary of genetic polymorphisms affecting Leflunomide metabolism.

Gene/Variant	Proposed Mechanism	Endpoint Studied	Evidence Strength	Main Limitations	Current Clinical Relevance	Reference
CYP2C19 (*2, *3, *4, *17; SNPs: C-163A, C-729T, T-739G)	Alters CYP-mediated bioactivation of leflunomide → affects teriflunomide formation	Teriflunomide levels; efficacy; toxicity	Low–moderate	Small studies; inconsistent findings; variability in metaboliser classification	No clinical application, potential biomarker	[37]
CYP1A2 *1F	Modifies CYP1A2 activity (environment-dependent, esp. smoking)	Enzyme activity; toxicity	Low	Strong environmental confounding (smoking); limited direct PK/PD data	No clinical application	[14,37,38]
CYP2C9 (*2, *3)	Reduced enzymatic activity → impaired metabolism	Toxicity (case reports)	Very low (case reports only)	Limited evidence; minor role of CYP2C9 in metabolism	No clinical relevance	[39]
CYP3A4	Minor role in metabolism	Not specified	Very low	Lack of relevant variants; minimal contribution	None	-
ABCG2 (C allele vs. A allele)	Efflux transporter → drug distribution and elimination	Teriflunomide levels; adverse effects; efficacy	Low–moderate	Inconsistent findings; functional impact unclear	No clinical application	[37,40,41]
ABCC2 (c.1446C>G; 24C>T)	Alters drug transport/excretion	Drug concentration; toxicity	Very low	No direct studies in leflunomide; extrapolation from other drugs	No clinical application	[45,46]
DHODH (SNP in exon 1; haplotype 2)	Target enzyme of teriflunomide → affects pharmacodynamics	Efficacy; toxicity; metabolite levels	Low–moderate	Limited studies; small cohorts	Potentially relevant but not implemented clinically	[29,43,47,48]
Cytokine genes (IL-1β, IL-6, TNF-α)	Modulation of inflammatory response	Treatment efficacy	Moderate	Single study; limited scope	No relevance	[36,49,50]

## Data Availability

No new data were created or analyzed in this study. Data sharing is not applicable to this article.

## Abbreviations

The following abbreviations are used in this manuscript:

ABC	ATP-binding cassette
ACPA/anti-CCP	Anti-citrullinated protein antibodies
bDMARD	Biologic disease-modifying antirheumatic drug
csDMARD	Conventional synthetic disease-modifying antirheumatic drug
DHODH	Dihydroorotate dehydrogenase
DMARD	Disease-modifying antirheumatic drug
HCQ	Hydroxychloroquine
IL-1Ra	IL-1 receptor antagonist
LEF	Leflunomide
MMP	Matrix metalloproteinases
MTX	Methotrexate
PGE2	Prostaglandin E2
RA	Rheumatoid arthritis
RF	Rheumatoid factor
SSZ	Sulfasalazine
tsDMARD	Targeted synthetic disease-modifying antirheumatic drug
UMP	Uridine monophosphate
